# Seroprevalence of transfusion transmissible viral infections among blood donors in a tertiary care hospital

**DOI:** 10.1186/1471-2334-12-S1-P26

**Published:** 2012-05-04

**Authors:** C Dhinesh Kumar, VK Panicker, RS Febe, R Krishnamoorthy

**Affiliations:** 1 Sri Ramachandra University, Department of Transfusion Medicine, Chennai, India

## Introduction

Timely transfusion of blood saves millions of lives but unsafe transfusion practices puts many people at risk of transfusion transmissible infection (TTI). TTI can exist as an asymptomatic disease in the host; so, donors must be screened for high risk behaviour related diseases.

## Aim

To estimate the seroprevalence of HIV, HBsAg and HCV among whole blood donors.

## Methodology

This study was conducted at Department of Transfusion Medicine, Sri Ramachandra University, Porur; Chennai during Jul-2010 to Jun-2011. The sample size in this study was 11,871. All samples were subjected to ELISA screening for anti-HIV 1 & 2, HBsAg and anti-HCV in addition to other mandatory tests.

## Results

Out of 11,871 samples, seroprevalence of HIV, HBsAg and HCV were estimated to be 0.05%, 1.58% and 0.13% respectively during the study period.

## Conclusion

Stringent screening of donors for TTI is crucial to ensure safe supply of blood and blood products. This study involved both voluntary and replacement donors. Prevalence of TTI was found to be low in females. Highly sensitive ELISA kits play a major role in detecting antibody to HIV, HCV and HBsAg. Furthermore, Nucleic Acid Testing (NAT) facilitates viral detection at a much earlier stage and therefore reduces the chances of window period infection transmission to 1 in a million.

